# The Origin of Sex

**Published:** 1900-12-22

**Authors:** 


### THE ORIGIN OF SEX.

THE inventors of great ideas should always read history, and before publishing their discoveries should make sure that the same track has not been followed by long processions of investigators and has not been already ascertained to lead to nowhere. At the last meeting of the Obstetrical Society Mr. E. Rumley Dawson read a paper on "The Essential Factor in the Causation of Sex: a New Theory of Sex," a paper which elicited from Dr. Blacker an expression of surprise that the author should claim originality for a thing—we suppose Dr. Blacker meant an idea—which dated back to 500 B.C. The novelty of the suggestion being thus disposed of, let us turn to the theory itself. Mr. Dawson said that normally the ovaries discharged ova independently of each other, probably working alternately. The proof of this was that the number of cicatricial depressions, the result of ruptured Graafian follicles, were nearly equal in each ovary, and together equalled the number of menstrual periods passed. Normal single pregnancy was the result of the fertilisation of an ovum from one ovary only, and the sex of the child depended upon which ovary supplied the ovum fertilised; if the right a male and if the left a female. To illustrate this, he quoted cases of ovariotomy where one ovary only was removed, the subsequent pregnancy giving a child corresponding in sex to the ovary which was not removed, and gave cases of tubal pregnancy where by operation a male fœtus was removed from the right tube, the corpus luteum being in the right ovary; and *vice versâ* where a female fœtus occupied the left tube, the corpus luteum being in the left ovary. In cases of twins and plural births the ovaries must act at or about the same time when the sex of the twins was different, while when twins were of the same sex it was shown that the ovary of one side might provide two ova. Again, those cases in which successive children were all of the same sex, or where, after a child of one sex was born, the remainder were of the opposite sex, were to be explained on the hypothesis of an original or an acquired unilateral sterility. The usual mixture of children as regards sex was, he said, to be explained by both ovaries usually being active, and thus supplying children of each sex, and he clinched the matter by the peculiarly striking remark that there were two ovaries only, and two sexes only. With gentle sarcasm the President (Mr. Alban Doran) brought the question to a practical test, and asked whether the author seriously believed that where a male heir was a matter of national importance the removal of a queen's left ovary would at least avoid disappointment? Dr. Blacker said it was impossible to believe that the male parent played no part in the determination of sex, and that the sex of the ovum was already settled at the time of its expulsion from the ovary; and he added that he could point to cases of unilateral ovariotomy and subsequent pregnancy which were in direct opposition to Mr. Dawson's assumption, a statement in which he was supported by other speakers. Finally he thought that any theory of sex put forward for acceptance must apply to the whole of the vertebrata. But birds had only one ovary. The common fowl had only one, the left ovary, and he asked, Would Mr. Dawson kindly explain where, according to his view, cocks came from? Such trivialities, however, Mr. Dawson declined to discuss. It was quite obvious, however, that the sense—shall we say the common sense—of the meeting was quite opposed to this old theory, notwithstanding all that Mr. Dawson could find to say in its favour.

